# Incidence and Prevalence of Childhood Obesity in Tehran, Iran in 2011

**Published:** 2017-10

**Authors:** Azadeh MOTTAGHI, Parvin MIRMIRAN, Katayoon POURVALI, Zhaleh TAHMASBPOUR, Fereidoun AZIZI

**Affiliations:** 1. Obesity Research Center, Research Institute for Endocrine Science, Shahid Beheshti University of Medical Sciences, Tehran, Iran; 2. Nutrition and Endocrine Research Center, Research Institute for Endocrine Science, Shahid Beheshti University of Medical Sciences, Tehran, Iran; 3. Dept. of Clinical Nutrition and Dietetics, School of Nutrition Sciences and Food Technology, Shahid Beheshti University of Medical Sciences, Tehran, Iran; 4. Dept. of Basic Sciences, School of Nutrition Sciences and Food Technology, National Nutrition and Food Technology Research Institute, Shahid Beheshti University of Medical Sciences, Tehran, Iran; 5. Endocrine Research Center, Research Institute for Endocrine Science, Shahid Beheshti University of Medical Sciences, Tehran, Iran

**Keywords:** Incidence, Prevalence, Obesity, Childhood, Iran

## Abstract

**Background:**

The aim of this study was to determine the incidence and prevalence of obesity in Tehranian children.

**Methods:**

Data from children participated in Tehran Lipid and Glucose Study (TLGS) were evaluated. Cut off points for definition of obesity was the CDC’s standard thresholds of the 95^th^ percentile and 85^th^ percentile for overweight. Prevalence, annual incidence of obesity, cumulative incidence over 10 year and the incidence density (cases per person-years) totally were calculated.

**Results:**

The annual incidence of obesity was 1.9 and 3.4% per year in the first 3 yr and decreased to 0.9 and 2.5% in the last 3 yr of follow-up in girls and boys, respectively. Incidence density rates were in line with cumulative incidence, with a rate of 20.7 per 1000 person-years between the ages of 5.6 and 15.5 yr. The prevalence of obesity was higher among children who had obese parents (*P*=0.03). Among all ages, across the quartiles of parents’ BMI, the prevalence of obesity increased with rising in paternal (*P*=0.001) or maternal BMI (*P*=0.004). Physical activity of mothers affected the prevalence of obesity in children at mean ages of 5.3 and 9.1 yr. Across quartiles of mother’s physical activity, from heavy to light, the prevalence of obesity increased among children, from 4.4% to 5.9% in children, aged 5.3 (*P*=0.02) and from 11.6% to 13.0% in children, aged 9.1 yr (*P*=0.03).

**Conclusion:**

Prevalence of obesity among children increased with age. Cumulative incidence of obesity in children who were overweight at baseline was much higher than other children.

## Introduction

Childhood obesity has become more prevalent in recent decades and according to WHO is predicted to become a major global health problem by 2020 (1). In the USA, the prevalence of high Body Mass Index (BMI) has dramatically increased since 1980. Incidence of obesity in American children with ages 5 to 14 yr old showed that 12.4% of these children were obese and 14.9%, entering the kindergarten were overweight (2). Prevalence of obesity and overweight increased with age, to the extent that in eight grade 20.8% and 17.0% of these children were obese and overweight; contrary to its prevalence, annual incidence of obesity decreased during kindergarten from 5.4% to 1.7%. The four-year incidence of obesity was higher between the ages of 7–11 yr, compared to ages of 11 and 15 yr (5.0% vs. 1.4%, respectively) (3). In 2010, 11.9% of children aged 2–19 were ≥ 97th percentile of the BMI-for-age growth charts, and 17% were ≥ 95th percentile (4). Similar trends have also been observed in Iranian children with reports indicating a prevalence of obesity in 6–12 yr old children of about 7%–16% (5, 6). In addition, overall, prevalence of elevated body mass index (16.6%) including obesity (9.1%) and overweight (7.5%) in children and adolescents, aged 10–18 yr, increased from 2003 to 2010 (7).

Obesity in childhood can be a predictor of later-life health problems such as diabetes, heart disease, and stroke (8) in addition to psychosocial problems such as poor self-esteem, body dissatisfaction, and social stigmatization (9–11). Different factors are involved in childhood obesity including genetic, environmental, lifestyle, socioeconomic status and parental characteristics. The relationship between overweight and obese parents and the risk of overweight in children has been well studied (12). Recently, parental body shape has also been reported as an important factor affecting childhood overweight (13). An inverse correlation has been found between childhood overweight and parental socioeconomic status viz. education, occupation and income level (14).

For the best results, studying obesity in children should include multiple points of measurement and well-mentioned weight changes over time. Unlike data on prevalence, no reports on the incidence of childhood obesity and overweight are available in Iran.

The purpose of the present study was to evaluate the prevalence and incidence of childhood obesity in Iranian children during four different phases according to the data from a national longitudinal study. We also examined the relationship between childhood obesity, parental characteristics, and physical activity.

## Materials and Methods

### Study population

Data from children participated in the Tehran Lipid and Glucose Study (TLGS) were evaluated. TLGS is an ongoing prospective, population-based study performed on representative sample of residents from district no. 13 of Tehran, indicated in Feb 1999. The aim of the TLGS is to determine prevalence of non-communicable disease risk factors and to develop healthy lifestyles to decrease these risk factors.

Written consents were obtained from all participants and the study protocol was approved by the ethics research council of the Research Institute for Endocrine Sciences, Shahid Beheshti University of Medical Sciences.

All children aged 3–7 yr at baseline were followed through sequential phases of data collection, in 1999 (first phase; mean age, 5.3 yr), 2001 (second phase; mean age, 9.1 yr), 2004 (third phase; mean age, 12.2 yr) and 2007 (fourth phase; mean age, 15.5 yr). Children were followed for 5716 person-years. Available data of studied children, including BMI of parents, physical activity level of parents and socioeconomic status of family were obtained. Information about physical activity was collected using the Lipid Research Clinic (LRC) questionnaire (15) and socioeconomic status was determined according to Four Factor Index of Social Status scale (16).

### Definition and evaluation of data

For categorization of participants into “over-weight” and “obese”, we used the 2000 Centers for Disease Control and Prevention (CDC) growth chart (17). Cut off points for definition of obesity was the CDC’s standard thresholds of the 95^th^ percentile and 85^th^ percentile for overweight. Of 901 subjects, 604 were not obese at baseline. Prevalence of obesity in each phase of study in boys and girls was determined. Incidence of obesity (occurrence of new case of obesity) for 604 children who were not obese at baseline was calculated. Due to varied interval between the study phases, we calculated the annual incidence of obesity by dividing the incidence by the duration times between study phases in years. Cumulative incidence over 9 yr and the incidence density (cases per person-years) totally and according to sex were determined. Cumulative incidence was calculated by dividing the number of new obesity cases to the number of person-years of follow-up, expressed as a rate per 1000 person-years. In order to demonstrate how the risk of incidence of obesity in overweight vs. normal weight children, risk ratio was determined by dividing the incidence of obesity in overweight to the incidence of obesity in normal weight children.

### Statistical analysis

We stratified the prevalence and incidence of obesity according to sex, parent’s BMI, parent’s physical activity and socioeconomic status of the family. To compare the risk of obesity between normal-weight and overweight children, we calculated risk ratios for the incidence of obesity in overweight children divided by the incidence in normal-weight children. Finally, we used logistic regression to determine odds ratios of obesity after 10 yr. Statistical analysis was performed using SPSS software (SPSS Inc., Chicago, IL, USA; ver. 20).

## Results

### Prevalence of obesity

Prevalences of obesity and overweight at phase 1 (mean age, 5.3 yr), were 5.2% and 6.9%, respectively. At the end of follow-up, (mean age, 15.5 yr) prevalence of obesity increased to 14.9% and 20.2% for obesity and overweight, respectively (Table 1). Prevalence of obesity had not increased between the ages of 12 and 15 yr.

**Table 1:** Prevalence of obesity among children according to socioeconomic status and parent’s BMI and phisical activity levels (1998–2010)

***Variable***	***No. of children***	***Prevalence of obesity (95% CI)***			
		**First phase (Mean age, 5.3 Yr)**	**Second phase (Mean age, 9.1 Yr)**	**Third phase (Mean age,12.2 Yr)**	**Fourth phase (Mean age,15.5 Yr)**
All children	901	5.2 (3.7–6.7)	12.4 (9.3–15.5)	15.3 (12.3–18.3)	14.9 (11.9–17.9)
Boys	451	5.5 (3.4–7.6)	16.0 (11.1–20.9)	16.9 (12.4–21.4)	18.4 (13.6–23.2)
Girls	450	4.9 (2.9–6.9)	9.0 (5.2–12.8)	13.8 (9.7–17.9)	11.5 (7.7–15.3)
Socioeconomic status *					
1	224	4.7 (2.0–7.4)	10.5 (5.1–15.9)	14.8 (9.3–20.3)	14.8 (9.1–20.5)
2	286	6.8 (3.8–9.8)	13.8 (8.5–19.1)	16.0 (10.7–21.3)	17.3 (11.8–22.8)
3	154	6.3 (2.3–10.3)	12.3 (4.8–19.8)	21.9 (13.6–30.2)	16.0 (8.6–23.4)
Mother’s BMI quartile^†^					
1	205	2.5 (0.3–4.7)	5.0 (0.7–9.3)	7.2 (2.7–11.7)	5.0 (1.01–8.9)
2	212	4.0 (1.3–6.7)	7.3 (2.1–12.5)	9.1 (4.2–14.0)	13.3 (7.4–19.2)
3	208	5.0 (2.0–8.0)	17.0 (9.6–24.4)	22.0 (14.9–29.1)	16.0 (9.7–22.3)
4	201	9.6 (5.5–13.7)	20.2 (12.5–27.9)	26.5 (18.7–34.6)	27.0 (18.7–35.3)
Father’s BMI quartile^‡^					
1	168	2.5 (0.1–4.9)	4.9 (0.2–9.6)	6.0 (1.4–10.7)	10.6 (4.7–16.5)
2	173	3.6 (0.8–6.4)	5.3 (0.8–9.8)	10.6 (4.9–16.3)	10.6 (4.7–16.5)
3	168	5.7 (2.1–9.3)	18.8 (10.5–27.1)	18.2 (11.0–25.4)	18.2 (10.6–25.8)
4	171	11.9 (7.0–16.8)	19.4 (11.4–27.4)	30.8 (22.4–39.1	23.3 (15.6–31.0 )
Mother’ s physical activity level					
Heavy	207	4.4 (1.6–7.2)	11.6 (5.7–17.5)	13.8 (7.9–19.7)	15.0 (8.8–21.2)
Moderate	87	4.7 (0.2–9.2)	12.8 (2.3–23.3)	24.1 (12.7–35.5)	25.0 (13.5–36.5)
Light	554	5.9 (3.9–7.9)	13.0 (8.9–17.1)	15.1 (11.3–18.9)	13.6 (9.8–17.4)
Father’s physical activity level					
Heavy	134	5.5 (1.5–9.5)	16.9 (7.8–26.0)	18.5 (10.0–26.9)	15.5 (7.8–23.2)
Moderate	105	5.9 (1.3–10.5)	14.3 (5.1–23.5)	19.1 (9.8–28.4)	14.9 (6.7–23.4)
Light	442	6.1 (3.8–8.4)	10.7 (6.7–14.7)	15.9 (11.7–20.1)	16.6 (12.2–21.0)

* Scores of socioeconomic status: 1 (8–30), 2 (31–36) and 3 (37–46)

† Mother’s BMI quartiles: 25 (23.7 kg/m^2^), 50 (26.6 kg/m^2^) and 75 (29.6 kg/m^2^)

‡ Father’s BMI quartiles: 25 (23.4 kg/m^2^), 50 (26.0 kg/m^2^) and 75 (28.4 kg/m^2^)

Data on phases 1, 2 and 4 showed that the highest prevalence of obesity belonged to children with modest socioeconomic status families. In three phases (mean age 12.2 yr) children from the wealthiest family had higher prevalence of obesity than others in all quartiles of socioeconomic status.

The prevalence of obesity was higher among children who had obese parents. Among all ages, across the quartiles of parents’ BMI, the prevalence of obesity increased with rising BMI in either parent. The greatest increases in the prevalence of obesity were seen in the children, aged 15.5 yr, in the upper quartiles of mother’s BMI and in children aged 12.2 in the upper quartiles of father’s BMI (27% and 30.8%, respectively).

Physical activity of mothers affected the prevalence of obesity in children with mean ages of 5.3 and 9.1 yr. Across the quartiles of mother’s physical activity, from heavy to light, the prevalence of obesity increased among these children, from 4.4% to 5.9% in children mean age 5.3 yr and from 11.6% to 13.0% in children mean age 9.1 yr.

**Fig. 1:**
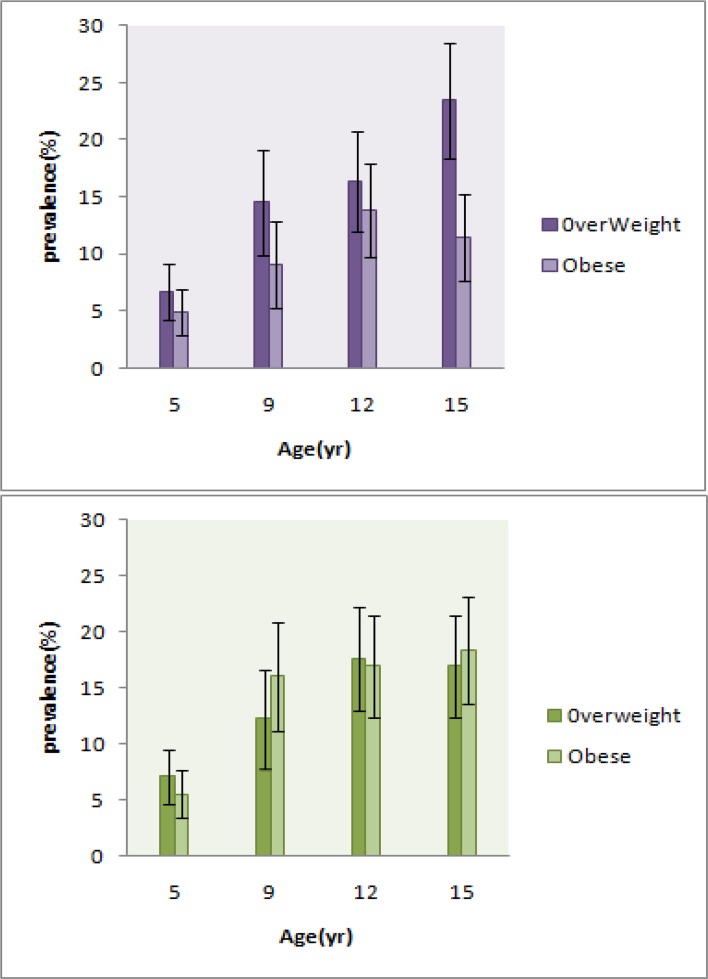
Trend of overweight and obesity prevalence in 10 yr. The black vertical lines represent 95% confidence intervals. A: Girls, B: Boys

**Fig. 2:**
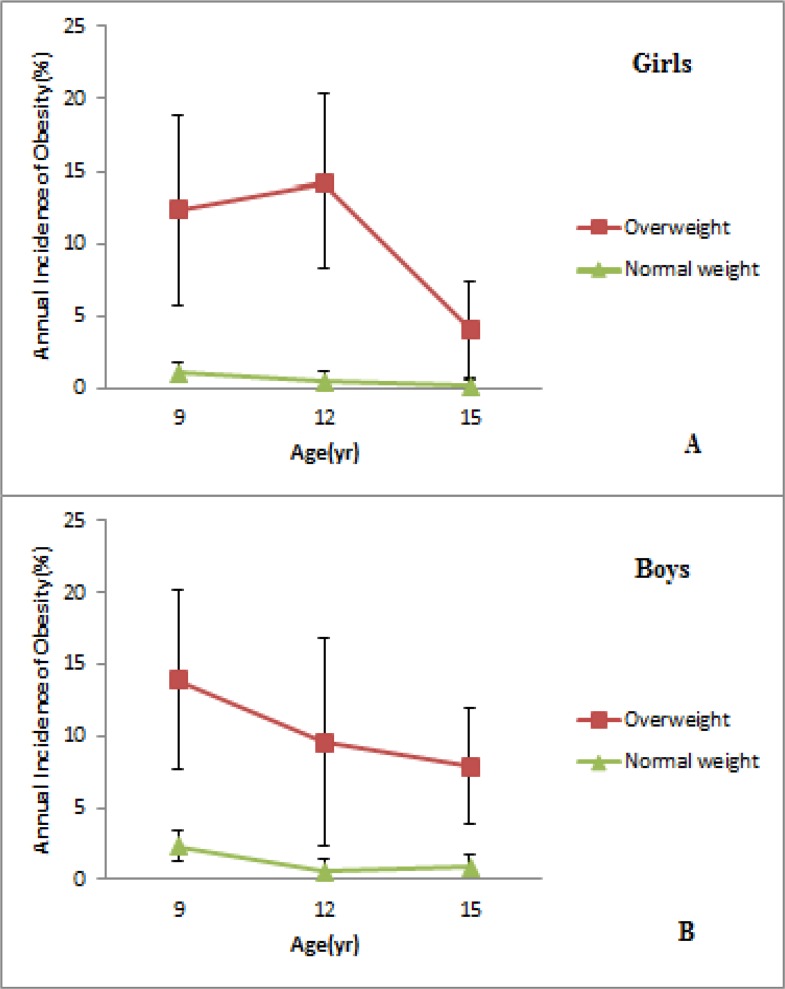
Annual incidence of overweight and obesity between phase 1 and phase 4 according to baseline weight. The black vertical lines represent 95% confidence intervals. A: Girls, B: Boys

Father’s physical activity had minor effect on the prevalence of obesity only in children at mean age 5.3 yr (from 5.5% to 6.1%). The highest prevalence of obesity in different levels of physical activity belonged to children who had mothers with moderate physical activity (24.1% at phase 3 and 25.0% at phase 4, Table 1). Fig. 1 and 2 illustrate additional information about the prevalence of overweight based on sex; as seen, the highest prevalence of overweight among girls and boys is around the ages of 15 and 12 yr respectively.

### Incidence of obesity

Incidence of obesity declined through the fourth phase in both sex, although obesity prevalence increased with age. The annual incidence of obesity among girls was 1.9% and reached 0.9% per year at the time between phases 2 and 4 (Fig. 2). For boys, the annual incidence of obesity per year decreased from 3.4% to 2.5% between phases 2 and 4 (Fig. 2).

During period between the ages of 5.3 and 15.5 yr, 21.3% of children (18.5% of girls and 24.3% of boys) became obese. Incidence density rates were in line with cumulative incidence, with a rate of 20.7 per 1000 person-years between the ages of 5.6 and 15.5 yr. At the end of study period, 51.3% of children who were overweight at baseline of study had become obese as compared with 18.8% of normal weight children. The annual incidence of obesity between phases 1 and 2, among children who were overweight was 12.3% in boys and 13.9% in girls and among normal weight, children were 1% in boys and 2.3% in girls. In line with these data, incidence density rates were 68.1% vs. 17.8% per 1000 person-years for overweight and normal weight children, respectively. Incidence of obesity among children, who were overweight at baseline, decreased with increasing age and between the ages of 12.2 and 15.5 yr, the annual obesity incidence was 6.1% (7.9% for boys and 4.1% for girls). Children who were overweight at baseline had a 4.5 fold risk of becoming obese as compared to those with normal weight by fourth phase of the study (Table 2).

**Table 2:** Cumulative incidence and risk ratio of obesity in 10 years, according to weight at baseline

***Variable***	***Normal weight at baseline (N= 557)***		***Overweight at baseline (N=47)***		***Risk ratio for overweight vs. normal weight (95% CI)***
	***Cumulative incidence (95% CI) %***	***P Value***	***Cumulative incidence (95% CI) %***	***P Value***	
All children	18.8(15.2–22.4)		51.3(35.6–67.0)		4.55(2.33–8.90)
Boys	22.0(16.6–27.4)	Refrence	50.0(28.1–71.9)	Refrence	3.55(1.34–9.02)
Girls	15.7(11.0–24.4)	0.08	52.6(30.7–74.5)	0.86	5.95(2.26–15.63)

Table 3 shows the cumulative incidence of obesity, at the end of 10 yr of follow-up; 24.7% of children from the modest socioeconomic status families, 21% of children from the poorest families and 19.3% of children from the wealthiest families became obese.

**Table 3:** Cumulative incidence of obesity in 10 years, according to weight at baseline across tertiles of socioeconomic status and phisical activity and quartiles of parents’ BMI

***Variable***	***Not obese at baseline (n=604)***		***Normal weight at baseline (n= 557)***	
	***Cumulative incidence (95% CI) %***	***P Value***	***Cumulative incidence (95% CI) %***	***P Value***
Socioeconomic status *				
1	21.0(14.3–27.7)	0.7	20.0(13.1–26.9)	0.4
2	24.7(18.2–31.2)	0.3	20.5(14.2–26.8)	0.4
3	19.3(10.8–27.8)	Reference	15.8(7.6–24.0)	Reference
Mother’s BMI quartiles^†^				
1	9.4(4.1–14.7)	Reference	8.2(3.1–13.3)	Reference
2	17.9(11.0–24.8)	0.6	16.8(9.7–23.9)	0.6
3	27.6(19.7–35.5)	0.001	25.0(17.0–33.0)	0.001
4	32.7(23.8–41.6)	<0.001	27.8(18.9–36.7)	<0.001
Father’s BMI quartiles^‡^				
1	13.3(6.6–20.0)	Reference	10.9(4.5–17.3)	Reference
2	13.7(7.0–20.4)	0.9	11.6(5.2–18.0)	0.9
3	28.0(18.9–37.1)	0.1	23.5(14.3–32.7)	0.03
4	32.7(23.8–41.6)	0.001	30.9(21.7–40.1)	0.001
Mother’s physical activity levels				
Heavy	22.1(14.7–29.5)	Reference	19.6(12.2–27.0)	Reference
Moderate	34.0(21.2–46.8)	0.1	30.6(17.7–43.5)	0.1
Low	19.5(15.0–24.0)	0.5	17.2(12.7–21.7)	0.5
Father’s physical activity levels				
Heavy	25.0(15.5–34.5)	Reference	20.8(11.4–30.2)	Reference
Moderate	19.4(9.6–29.2)	0.4	17.5(7.6–27.4)	0.6
Low	22.2(17.1–27.3)	0.6	19.6(14.5–24.7)	0.8

* Scores of socioeconomic status: 1 (8–30), 2 (31–36) and 3 (37–46)/

† Mother’s BMI quartiles: 25 (23.7 kg/m^2^), 50 (26.6 kg/m^2^) and 75 (29.6 kg/m^2^)/

‡ Father’s BMI quartiles: 25 (23.4 kg/m^2^), 50 (26.0 kg/m^2^) and 75 (28.4 kg/m^2^)

Cumulative incidence of obesity increased significantly with rise in the mothers’ BMI (9.4% vs. 32.7%) and fathers’ BMI (13.3 vs. 32.7%).

The highest cumulative incidence based on mothers’ physical activity was 34% and was seen among in children, whose mothers had moderate physical activity; however, parents’ physical activity had no significant effect on the incidence of obesity. As compared with those who were not obese at baseline, the incidence of obesity among children who had normal weight was slightly lower across the tertiles of socioeconomic status and physical activity and quartiles of parents’ BMI.

## Discussion

Findings of this study show that the prevalence of obesity increased by age and reached 14.9% during 10 yr, compared to 5.2% at baseline. The maximum slope of increase in obesity prevalence was seen in children, aged 5 to 9 yr (5.2% to 12.4%).

Other studies in Iranian children also report similar prevalences. Overall, 17% of school boys between 14 and 15 yr, were overweight and 2% of the same population were obese (18). Moreover, a cross-sectional study reported that 18.8% and 14.3% of school children (aged 7–12 yr) were overweight and obese, respectively and the odds ratio for becoming obese was 1.26 with each year of age increase (19).

The results of two Iranian national surveys of a surveillance program were compared, CASPIAN-I (2003–2004) and CASPIAN-III (2009–2010) and the overall prevalence of increased BMI (16.6%), including obesity (9.1%) and overweight (7.5%) increased from 2003 to 2010 in school students (7). Although the aforementioned studies were carried out in different parts of Iran, almost similar result was obtained which could be the result of similar diet and lifestyle habits rather than geographical or the possible genetic differences. In contrast, the annual incidence of obesity dropped between phases 2 and 4 in both genders indicating that most children who were obese or overweight at the end of the study, had been obese or overweight at a younger age or at baseline.

Children aged 7–15 yr, incidence of obesity was higher between 7–11 yr than between 11–15 yr (5.0% vs. 1.4%) (3). In addition, London students were followed up aged 11–12 for five yr and suggested the incidence of obesity was low between ages 11 and 15 yr and there was no decrease in the proportion of healthy students indicating persistent obesity is established before age 11 (20). Contrary to the hypothesis that most excess weight gain occurs in early childhood in English children, excess weight gain was substantial in mid-childhood, between the ages of 5–9 yr (21).

The decrease in the incidence of obesity at later phases of this study could reflect changes in lifestyle and the increase of nutrition-related information obtained from social media and at school as the children grows. About half of the over-weight children became obese by the end of study and these children had 4.5 fold chance of becoming obese as compared with normal-weight children. Overall, 6807 American children, were followed for 10 yr; 45% of children, between 5 and 14 yr of age, who became obese, were over-weight at baseline; the prevalence of both obesity and overweight increased during this time, however, the annual incidence of obesity decreased from 5.4% during kindergarten to 1.7% between fifth and eighth grade (2). Similarly, overweight and obesity before age 11 are likely to persist seven years later (20). Therefore, the age of onset of overweight or obesity could serve as a predictor of adulthood obesity.

The increase in both prevalence and cumulative incidence of obesity was associated with increasing parent’s BMI. A similar pattern and OR of 1.18 and 1.15 for one unit increase in mother and father’s BMI in obese children (19). In our study, the highest increase in the prevalence was seen in phases 3 and 4 (mean ages of 12.2 and 15.5 yr) in upper quartiles of father’s and mother’s BMI, respectively. In a total of 1189 school children, aged 12–14 yr, the father’s BMI was significantly associated with son’s BMI (OR: 2.02) and daughter’s BMI (OR: 1.59), whereas the mother’s BMI was significantly associated with the daughter’s BMI only (OR: 0.51) (22).

Subjects with both parents overweight, compared with those with only one or neither parent over-weight had more chance of becoming overweight (23). However, the separate role of paternal and maternal BMI on child obesity is not clearly understood. Different studies have shown the father’s role in the child’s BMI (24, 25), but the effect of parent’s BMI on the child’s is independent of each other (26). Totally, 2025 sets of parents and their children (7–13 yr) were evaluated and reported that the odds ratio of overweight was 2.26 for children with overweight father, normal weight mother, 2.71 when father was normal weight, mother was overweight, and 4.36 for both father and mother overweight group, respectively, compared with children with nonoverweight parents (27). Therefore, parental BMI imposes important effect on childhood body weight.

This relationship could also be a result of both genetic and the lifestyle factors, such as dietary and activity patterns that a family share, parental education level and their ability to change unhealthy eating behavior in family.

The prevalence of obesity in phases 1 and 2 (mean age 5.5 and 9.1) was inversely associated with mother’s physical activity, while the father’s physical activity impact was minor. Interestingly, in older children (phase 3 and 4) mothers with moderate physical activity had the highest prevalence of obese children, and the highest cumulative incidence also belonged to these mothers.

Research has shown a positive association between parental and child’s activity and the odds ratio for children to participate in sport was 3.9 (girls) and 8.8 (boys) when both parents were active (28, 29). Besides, low parental habitual physical activity scores were suggested as significant predictors of childhood overweight (30).

The reason why moderate activity of mother was associated with child obesity is not clear.

The highest prevalence of obesity is mostly in families with modest socioeconomic status. In Korean children there was an inverse association between parental socioeconomic status and the child weight (13) which is in agreement with our current results as only at the mean age of 12.2 yr (phase 3), obesity was associated with the wealthiest families. Adult obesity in developing countries was the disease of the socioeconomic elite, however, it is now shifting toward the low socioeconomic groups (31). Different SES groups are at different risks, and the relationship between obesity and SES varies across countries (32).

Families with lower socioeconomic status usually have limited access to fast food and other high-calorie food and lower prevalence of obesity in these families is justifiable; on the other hand, lower prevalence of childhood obesity in wealthiest families as compared to families with modest socioeconomic status can be due to higher education levels and knowledge of parents regarding healthier foods, healthy eating habits and easier access to physical activity and healthy foods.

Longitudinal design, access to data about socioeconomic status, parent’s BMI and physical activity levels are strengths of present study. One of the major limitations of this study is no information about weight at birth and trend of weight gain before entering into survey. These data can affect interpreting of results and final decisions.

## Conclusion

Children’s weight at preschool age is the main factor for determination of obesity at adolescent and school age and should be considered in national strategies program for prevention of childhood obesity. Further research is necessary to elucidate all other contributing genetic and environmental factors that could lead to overweight in adolescence.

## Ethical considerations

Ethical issues (Including plagiarism, informed consent, misconduct, data fabrication and/or falsification, double publication and/or submission, redundancy, etc.) have been completely observed by the authors.
